# Global hotspots and emerging trends in 3D bioprinting research

**DOI:** 10.3389/fbioe.2023.1169893

**Published:** 2023-05-25

**Authors:** Zhiyu Ding, Ning Tang, Junjie Huang, Xu Cao, Song Wu

**Affiliations:** ^1^ Department of Orthopaedics, Third Xiangya Hospital of Central South University, Changsha, Hunan, China; ^2^ Institute of Basic Medicine and Cancer (IBMC), Chinese Academy of Sciences, Hangzhou, Zhejiang, China

**Keywords:** 3D bioprinting, tissue engineering, bio-ink, hydrogels, extrusion-based bioprinting, bibliometrics

## Abstract

Three-dimensional (3D) bioprinting is an advanced tissue engineering technique that has received a lot of interest in the past years. We aimed to highlight the characteristics of articles on 3D bioprinting, especially in terms of research hotspots and focus. Publications related to 3D bioprinting from 2007 to 2022 were acquired from the Web of Science Core Collection database. We have used VOSviewer, CiteSpace, and R-bibliometrix to perform various analyses on 3,327 published articles. The number of annual publications is increasing globally, a trend expected to continue. The United States and China were the most productive countries with the closest cooperation and the most research and development investment funds in this field. Harvard Medical School and Tsinghua University are the top-ranked institutions in the United States and China, respectively. Dr. Anthony Atala and Dr. Ali Khademhosseini, the most productive researchers in 3D bioprinting, may provide cooperation opportunities for interested researchers. Tissue Engineering Part A contributed the largest publication number, while Frontiers in Bioengineering and Biotechnology was the most attractive journal with the most potential. As for the keywords in 3D bioprinting, Bio-ink, Hydrogels (especially GelMA and Gelatin), Scaffold (especially decellularized extracellular matrix), extrusion-based bioprinting, tissue engineering, and *in vitro* models (organoids particularly) are research hotspots analyzed in the current study. Specifically, the research topics “new bio-ink investigation,” “modification of extrusion-based bioprinting for cell viability and vascularization,” “application of 3D bioprinting in organoids and *in vitro* model” and “research in personalized and regenerative medicine” were predicted to be hotspots for future research.

## 1 Introduction

Three-dimensional (3D) bioprinting is a technology that enables the 3D printing of various cells, biocompatible materials, and supporting components into complex 3D functional living tissues (Murphy and Atala, 2014). This technique allows to print cell-embedded biomaterials in a layer-by-layer manner using computer-controlled automated dispensing systems (Matai et al. 2020). This method enables single or multiple cells to be arranged in a specified form in biomaterials, thereby realizing the construction of functional 3D tissues with cells (Derby, 2012; Tasoglu and Demirci, 2013; Ozbolat, 2015).

The advantage of 3D bioprinting technology lies in its high printing resolution, which allows for precise allocation of cells, matrix, biomolecules, and biological materials to mimic the natural tissue structure (Jana and Lerman, 2015). Traditional tissue engineering techniques can only control volume characteristics through fiber bonding, freeze-drying, solvent casting and electrospinning, et al. but cannot customize pore size, shape, network, internal structure, and topological structure. Besides, traditional tissue engineering methods also cannot easily achieve specific requirements for porosity. With the assistance of computer-aided design (CAD) technology, 3D bioprinting can efficiently and economically produce complex 3D structures ranging from nanoscale to microscale (Huang et al. 2017). Due to the superior performance of the 3D bioprinting technology, it has been a research hotspot in various fields of tissue engineering, such as transplant and regenerative medicine, as well as disease model construction. For instance, several types of bio-ink and printing processes have been developed for applications in tissue engineering (Liang et al. 2022; Luo et al. 2022; Murab et al. 2022; Sonaye et al. 2022; Thangadurai et al. 2022). In addition, 3D bioprinting has been widely used in artificial organ fabrication and tissue regeneration (Ramadan and Zourob, 2020; Jain et al. 2022; Panda et al. 2022; Pourmasoumi et al. 2022; Wang et al. 2023). Moreover, owing to its unique advantages, 3D bioprinting plays a key role in the construction of common disease *in vitro* models and organoids (Fan et al. 2019; Guagliano et al. 2022; Joddar et al. 2022; Jubelin et al. 2022; Neufeld et al. 2022; Shakir et al. 2022).

Over the past 20 years, there have been continuous breakthroughs in 3D bioprinting research, especially in the modification of bio-ink and printing technology, as well as their applications. Therefore, it is necessary to discuss the progress in this field. Bibliometrics is an interdisciplinary science of quantitative analysis of all knowledge using mathematical and statistical means, which can estimate the structure and development of specific scientific disciplines (Tang et al. 2021b; Tang et al. 2021c; Ding et al. 2022). Although there have been related studies published in the field of 3D bioprinting (Naveau et al. 2017; Santoni et al. 2022), the exponentially growing number of articles requires the latest bibliometric analysis to help researchers in this field understand the latest hot topics.

To further understand the development of 3D bioprinting, in this study we propose to use bibliometric methods to highlight the characteristics of articles on 3D bioprinting, especially in terms of research hotspots and focus. We hope our research can provide a more comprehensive understanding for researchers in the field of 3D bioprinting about the current state and future research development trends.

## 2 Materials and methods

### 2.1 Data collection

As an influential citation-based database, the Web of Science Core Collection (WoSCC) database has been widely used in bibliometric studies (Zhang et al. 2020; Zhu et al. 2020; Cheng et al. 2022). We performed a comprehensive online search in this database on the last day of November 2022 to reduce bias caused by database updates. We use “topic” as the search scope, which mainly includes the title, abstract, keyword, and other relevant elements sections of the article. The search formula was as follows: [#1: TS = (“bioprint*” OR “bio-print*”); #2: TS = (3D OR “3 dimensions” OR “three dimensions” OR “three dimensional” OR “3 dimensional”); Final dataset: #1 AND #2]. Language was limited to English. No time and publication type restrictions were used in this search. A total of 4,823 search results were retrieved in the initial search. Then, two researchers (ZYD and NT) manually examined the information of the retrieved documents, such as titles, abstracts, and the full text. Publications not related to 3D bioprinting were excluded (Supplementary Table S1). Finally, a total of 3,327 manuscripts were identified and included for further analysis. The flow chart is illustrated in Supplementary Figure S1.

### 2.2 Data analysis and visualization

Several software are frequently used in bibliometric analysis, including VOSviewer, CiteSpace, and HistCite (Zhang et al. 2021a). Two software and one online site were applied to the following analysis in this study.

VOSviewer 1.6.18 is a widely used software for constructing knowledge-maps based on a co-occurrence matrix (Farzanegan et al. 2017). The general information was standardized before analysis. Standardized author keywords that were manually paraphrased by the authors reduced the bias better. For example, we included “three-dimensional bioprinting” under “3d bioprinting” (Romero and Portillo-Salido, 2019). In terms of national information, we classified Taiwan as China (Gao et al. 2019). This bibliometric tool provided co-occurrence/co-citation/co-authorship maps of the restricted data. Generally, the larger the size of the diverse nodes/words, the more times they occur. The color line between two nodes represents the degree of connection. The thicker the line, the more widespread the cooperation (Shi et al. 2021). The color of the nodes indicates different average publication years.

CiteSpace 6.1. R2 is another bibliometric software for creating visualization maps based on the data retrieved from the database (Cheng et al. 2022; Feng et al. 2022). Several knowledge-based visual maps were constructed, such as citation bursts map, timeline view, and dual-map overlay. The basic parameter settings were as follows: years per slice (1), pruning (minimal spanning tree and pruning sliced nets), and inclusive standards (top N = 50), others were set as default (Zhang et al. 2021a). In the co-occurrence of countries/regions and institutions maps, node colors changed from blue to red representing the publication years from 2007 to 2022.

Moreover, an online platform (https://bibliometric.com/app_v0) was used for the comparison analysis of the annual number of papers among the top 10 countries. GraphPad Prism 8 and Microsoft Office Excel 365 were respectively used to draw a column chart and provide a descriptive analysis for annual publications. Impact factor (IF) scores were extracted from 2021 Journal Citation Reports (JCR).

## 3 Results

### 3.1 Publication outputs and trends

**FIGURE 1:**
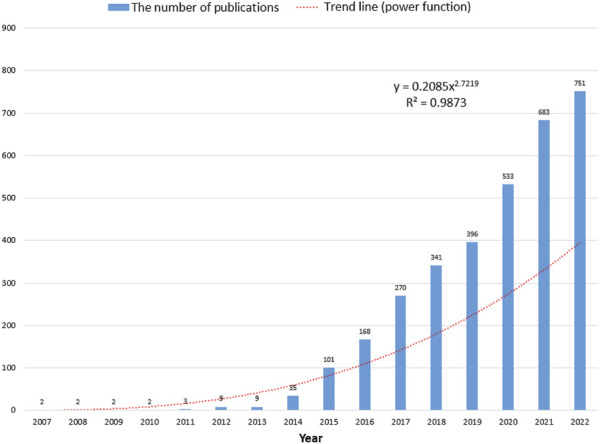
The corresponding number of annual publications regarding 3D bioprinting from 2007 to 2022.

Based on the search strategy, a total of 3,327 papers were identified. The number of annual publications regarding 3D bioprinting is illustrated in Figure 1. From 2007 to 2022, the number of publications per year showed a rapid growth trend, reaching over 100 publications for the first time in 2015. To evaluate the change trend, a power function (y = 0.2085 × 2.7219, *R* ^2^ = 0.9873) of the trend was created, where X represents the year and Y indicates the amount of annual publications.

### 3.2 Most prolific countries/regions, funding agencies and institutions

**FIGURE 2:**
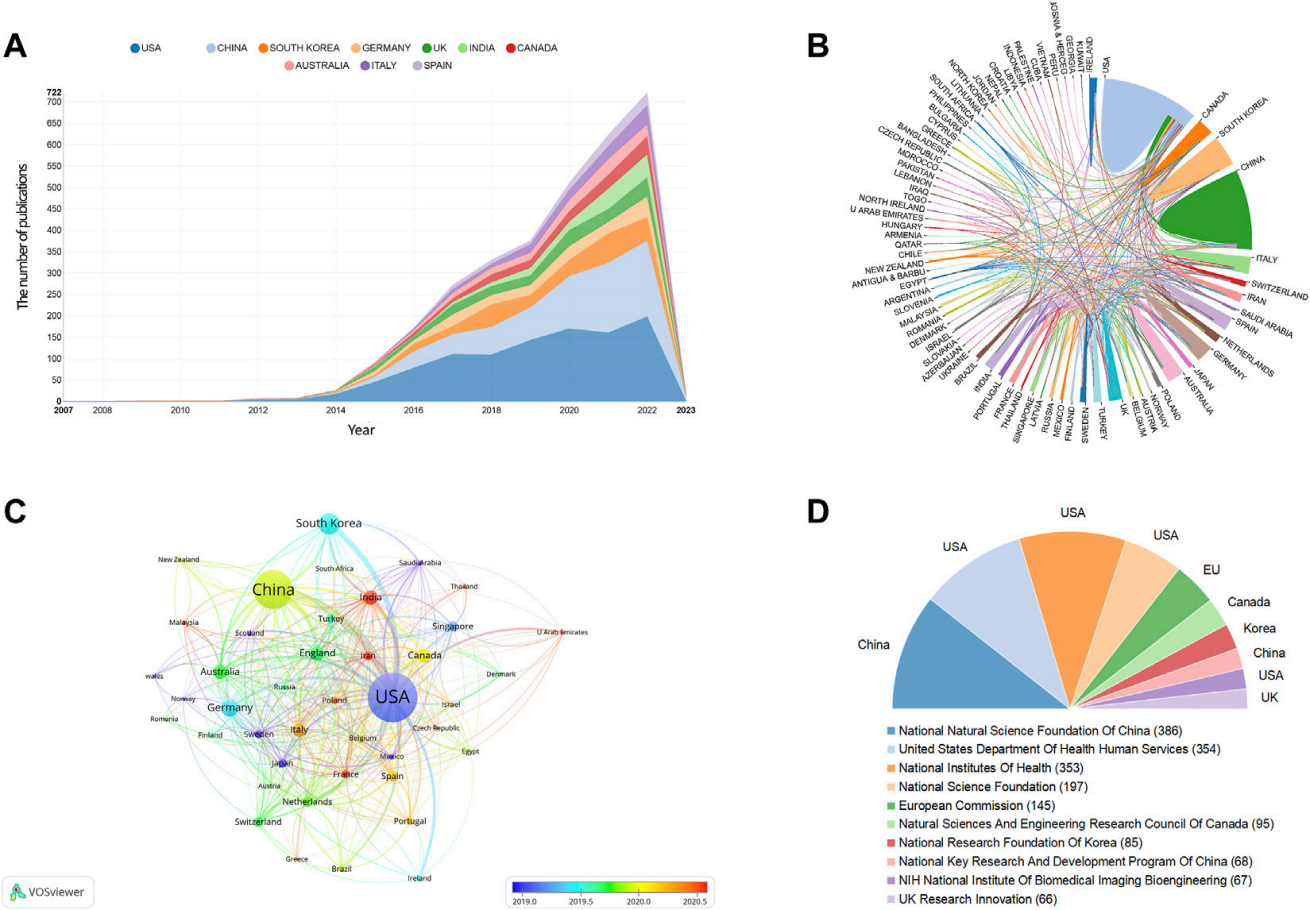
The distribution of counties/regions and funding agencies in 3D bioprinting research. **(A)** The number of publications per year of the top 10 countries. **(B)** The international cooperation analysis. The line between two counties indicates cooperative relationship. **(C)** The overlay visualization of co-authorship. The size of notes/words represent the total publications of a countries. The color line between two nodes represents the degree of co-authorship. The color of the nodes indicated different average publication year. **(D)** The top ten frequent funding agencies and corresponding countries.

All publications regarding 3D bioprinting were published by 2,733 institutions in 83 countries/regions in total. The top 10 prolific countries and institutions are listed in Table 1. Figure 2A illustrates the annual number of publications of the top 10 countries from 2007 to 2022. The most productive country is United States (*n* = 1,069), followed by China (*n* = 733), South Korea (*n* = 288), Germany (*n* = 210), and India (*n* = 166). As shown in Table 1, the United States has the highest centrality of 0.34 among all the countries, which is much higher than that of other countries/regions. The international cooperation analysis is shown in Figure 2B, in which the line between two counties indicates a cooperative relationship. The United States has the most cooperation with other countries in 3D bioprinting. The overlay visualization map among the countries is illustrated in Figure 2C. Countries/regions with a minimum of 10 publications were identified. The top 10 funding agencies, of which four are based in the United States, are summarized in Figure 2D. The National Natural Science Foundation of China is the most frequent funding source.

**TABLE 1:** The top 10 countries and institutions in 3D bioprinting research.

Rank	Countries	Count	Centrality	Institution	Count	Centrality
1	USA	1,069	0.34	Harvard Med Sch (USA)	86	0.14
2	China	733	0.07	Tsinghua Univ (China)	75	0.12
3	South Korea	288	0.09	Chinese Acad Sci (China)	74	0.08
4	Germany	210	0.13	Zhejiang Univ (China)	69	0.07
5	India	166	0.11	Wake Forest Sch Med (USA)	56	0.07
6	England	160	0.14	Shanghai Jiao Tong Univ (China)	45	0.11
7	Canada	153	0.07	Nanyang Technol Univ (Singapore)	45	0.03
8	Australia	152	0.05	Univ Calif Los Angeles (USA)	39	0.18
9	Italy	141	0.12	Univ Wollongong (Australia)	35	0.1
10	Spain	104	0.12	Univ Calif San Diego (USA)	35	0.05

**FIGURE 3:**
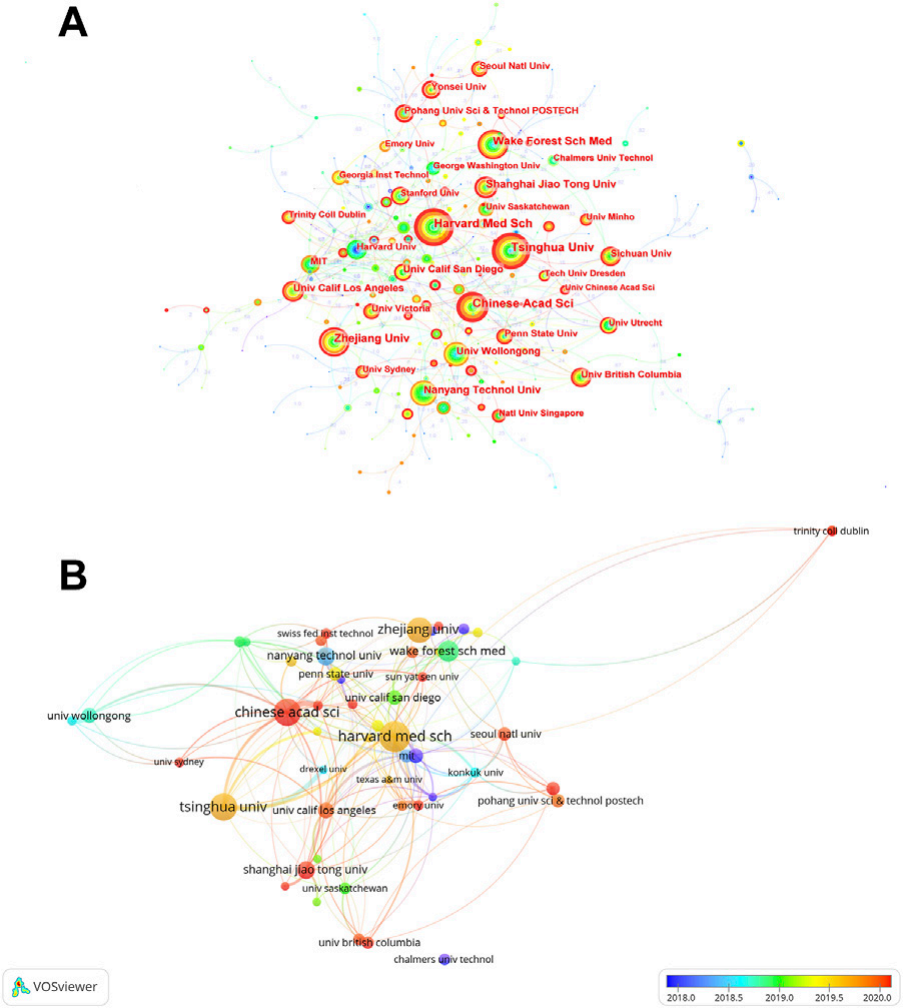
The distribution of institutions in 3D bioprinting research. **(A)** Co-occurrences of institutions. The notes represent corresponding institution. The size of notes/words represent the total outputs of one institution. The value between lines indicates the cooperation degree among two connected institutions. **(B)** Overlay visualization map of co-authorship.

The co-occurrences of institutions are presented in Figure 3A and the top 10 prolific institutions are listed in Table 1. Harvard Medical School (*n* = 86) is the institution with the largest number of publications, followed by Tsinghua University (*n* = 75), Chinese Academy of Sciences (*n* = 74), Zhejiang University (*n* = 69), and Wake Forest School of Medicine (*n* = 56). University of California, Los Angeles, has the highest centrality of 0.18. Of the top 10 most prolific institutions, four were from China and four were from United States. Moreover, an institution co-authorship analysis is shown in Figure 3B. Based on the color gradient, the newer average publication year of institutions, such as Chinese Academy of Sciences, Shanghai Jiao Tong University, and Yonsei University, was assigned the color red.

### 3.3 Analysis of influential authors and Co-cited authors

**FIGURE 4:**
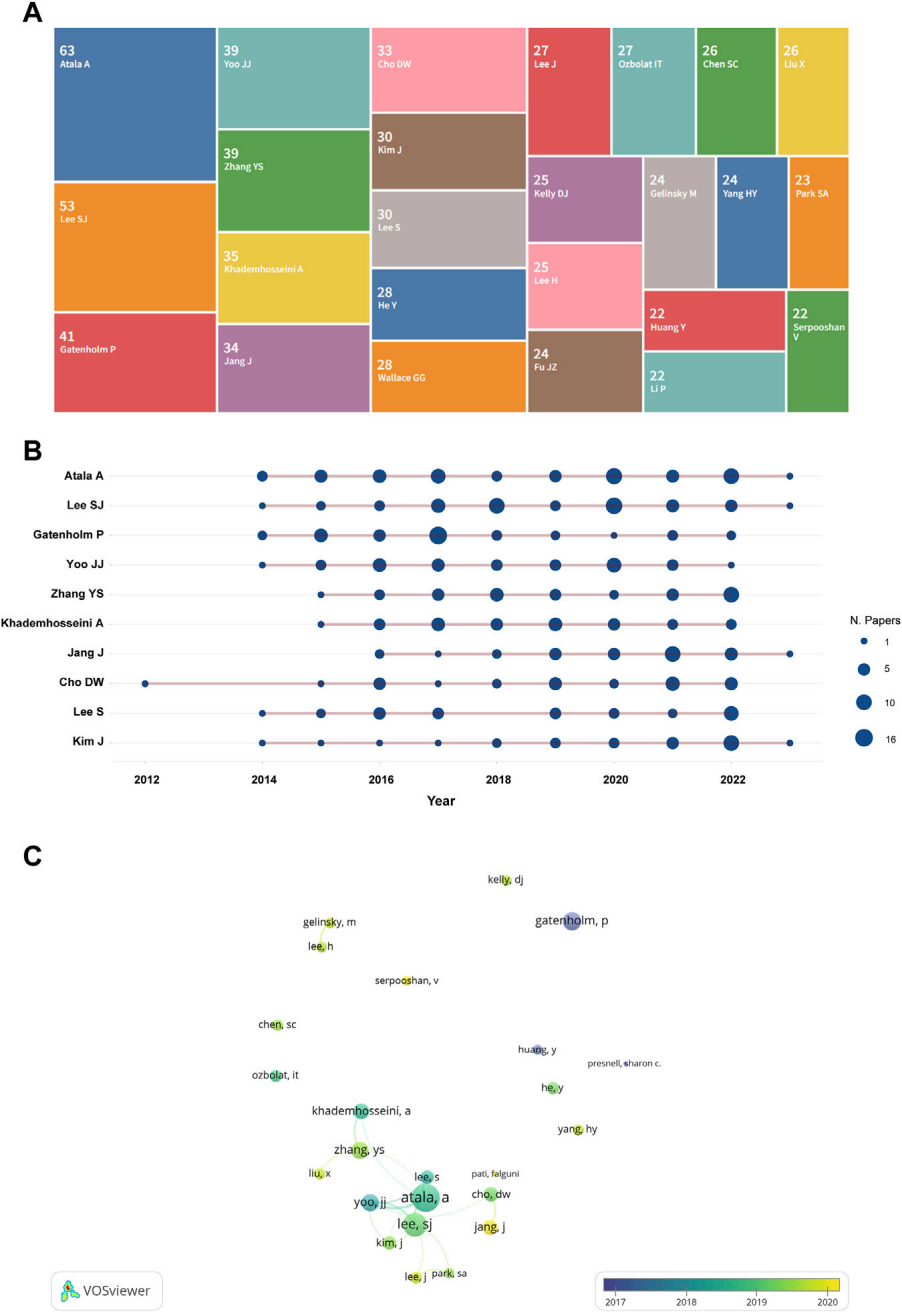
The overlay visualization of authors in 3D bioprinting research. **(A)** Tree map of the top 25 productive authors. The number above the name represent the amount of the publications. **(B)** Top 10 authors’ outputs over time. The size of the deep blue circle indicates the number of papers (N. papers). **(C)** The visualization of the top 25 prolific author relationships.

The top 25 most prolific authors are shown in Figure 4A. Among them, the information of the top 10 most productive authors is illustrated in Figure 4B and Table 2. Atala A (*n* = 63) contributed the largest number of papers, followed by Lee SJ (*n* = 53), Gatenholm P (*n* = 41), Zhang YS (*n* = 39), and Yoo JJ (*n* = 39). These and the rest of the authors are listed in Table 2. Figure 4B illustrates the annual publications of these authors. Published articles are mainly from 2014 to 2022. The visualization of the top 25 prolific author relationships was shown in Figure 4C. We can notice more collaborations between Atlas A, Lee SJ, Yoo JJ, Kim J, Lee J, Zhang YS, Khademhosseini A, Cho DW, Park SA and Jiang J, but there almost no collaboration among other highly productive authors.

**TABLE 2:** The top 10 most productive authors of 3D bioprinting research.

Rank	Authros	Count	Average citations per papers	H-index	Co-cited authors	Co-citations
1	Atala A	63	113.14	20	Murphy SV	1,139
2	Lee SJ	53	73.49	23	Ozbolat IT	806
3	Gatenholm P	41	36.27	10	Skardal A	691
4	Zhang YS	39	97.18	22	Kolesky DB	650
5	Yoo JJ	39	75.97	16	Pati F	589
6	Khademhosseini A	35	122.97	27	Ouyang LL	566
7	Jang J	34	20.44	12	Xu T	530
8	Cho DW	33	51.42	16	Mironov V	529
9	Kim J	30	7.10	10	Ng WL	510
10	Lee S	30	5.67	7	Kang HW	497

**FIGURE 5:**
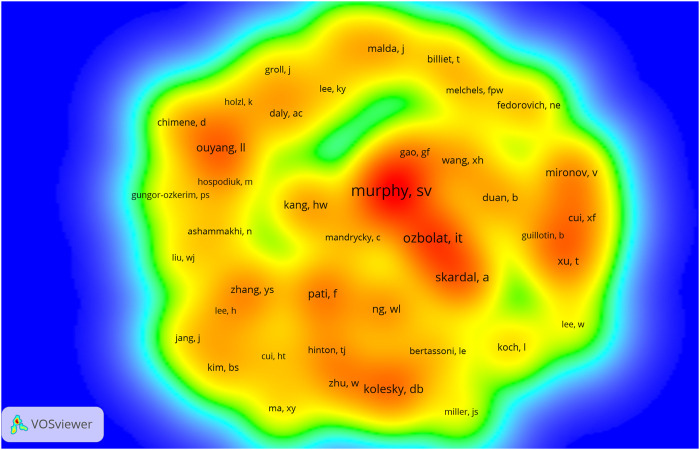
The density visualization of co-cited authors in 3D bioprinting research. The size of the circle/word are positively related to the number of co-citations.

The density maps of co-cited authors based on co-citations are shown in Figure 5, co-cited authors with ≥250 co-citations were included. The authors with the most co-citations are Murphy SV (*n* = 1,139), followed by Ozbolat IT (*n* = 806), Skardal A (*n* = 691), Kolesky DB (*n* = 650), and Pati F (*n* = 589). These and the rest of the top 10 co-cited authors are listed in Table 2.

### 3.4 Most active journals

**FIGURE 6:**
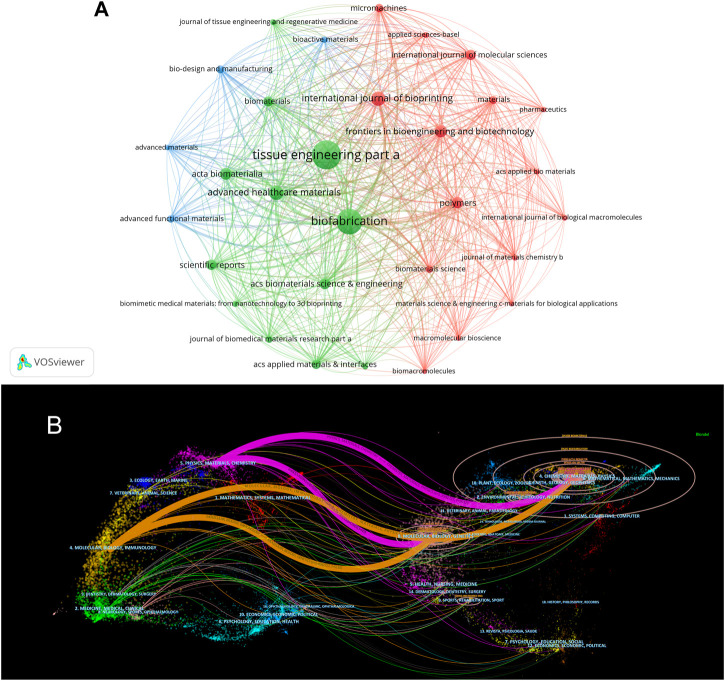
The visualization of journals in 3D bioprinting research. **(A)** Network visualization map of journals analysis. **(B)** Dual-map overlay of journals. The citing journals are on the left, the cited journals are on the right. The coloured path represents citation association of journals.

The articles included in the analysis were published in 752 journals. Of these, 33 published a minimum of 20 papers and were included and visualized in Figure 6A. The top 10 most prolific journals and their basic information are shown in Table 3. The journal that published the greatest number of papers is Tissue Engineering Part A (*n* = 225), followed by Biofabrication (*n* = 191), Advanced Healthcare Materials (*n* = 82), International Journal of Bioprinting (*n* = 81), and Frontiers in Bioengineering and Biotechnology (*n* = 65). All the top 10 most productive journals had an IF (2021) of >4. Seven of these journals were categorized in the Q1 JCR division.

**TABLE 3:** The top 10 most productive journals in 3D bioprinting research.

Rank	Journal	Count	IF 2021	JCR quartile 2021
1	Tissue Engineering Part A	225	4.080	Q2
2	Biofabrication	191	11.061	Q1
3	Advanced Healthcare Materials	82	11.092	Q1
4	International Journal of Bioprinting	81	7.422	Q1
5	Frontiers in Bioengineering and Biotechnology	65	6.064	Q1
6	Acta Biomaterialia	61	10.633	Q1
7	Polymers	57	4.967	Q1
8	ACS Biomaterials Science & Engineering	55	5.395	Q2
9	Scientific Reports	52	4.997	Q2
10	International Journal of Molecular Sciences	49	6.208	Q1

The dual-map overlay of journals created using CiteSpace is shown in Figure 6B. Four citation paths were recognized, indicating that the studies published in Molecular/Biology/Immunology or Physics/Materials/Chemistry journals were primarily cited by the research published in Molecular/Biology/Genetics or Chemistry/Materials/Physics journals.

### 3.5 Co-cited references and reference burst

Table 4 summarizes the top 10 most co-cited references related to 3D bioprinting research. The publication year of these papers was between 2014 and 2019. Two of these papers’ co-cited references were cited over 300 times. Nature Biotechnology and Biotechnology Advances both have two publications in the list. Of the top ten most cited references, six are review articles and four are research articles. The six review articles mainly summarize the applications and advances of bioinks (Hölzl et al. 2016; Hospodiuk et al. 2017; Gungor-Ozkerim et al. 2018) and 3D bioprinting technology (Murphy and Atala, 2014; Mandrycky et al. 2016; Ozbolat and Hospodiuk, 2016). Regarding the four research articles, Kolesky DB firstly proposed a bioprinting method that can be used to manufacture 3D tissue structures filled with vascular systems, multiple types of cells, and extracellular matrix, this structure opens new avenues for basic research in drug screening and wound healing, angiogenesis, and stem-cell niches (Kolesky et al. 2014). This team also proposed a new 3D bioprinting method that can create thick human tissue filled with engineered extracellular matrix, embedded vascular systems, and multiple types of cells, the proposal of this new method greatly promotes the development of using 3D bioprinting technology to construct human tissues for *in vitro* and *in vivo* applications (Kolesky et al. 2016). In 2006, Atala A led his team to develop an integrated tissue–organ printer (ITOP) that can fabricate stable, human-scale tissue constructs of any shape, they also demonstrated the use of the 3D bioprinter to manufacture mandibles, cranial bones, cartilage, and skeletal muscle. ITOP may be the prototype of various types of 3D bioprinters currently available, and its emergence has made it possible to construct more complex tissues and solid organs (Kang et al. 2016). In 2019, (Lee et al. 2019) proposed a method named FRESH (Freeform Reversible Embedding of Suspended Hydrogels) to 3D bioprint collagen, and used this method to construct heart tissue with patient-specific anatomical structures. This technology achieved gelation through pH control and has higher resolution (20-μm), making an important step in the study of using 3D bioprinting to construct organ tissues.

**TABLE 4:** The Top 10 Most Co-cited References in 3D bioprinting research.

Rank	Title (publication year)	First author	Journal	Co-citations
1	3D bioprinting of tissues and organs (2014)	Murphy SV	Nature Biotechnology	470
2	A 3D bioprinting system to produce human-scale tissue constructs with structural integrity (2016)	Kang HW	Nature Biotechnology	398
3	Current advances and future perspectives in extrusion-based bioprinting (2015)	Ozbolat IT	Biomaterials	269
4	Bioinks for 3D bioprinting: an overview (2018)	Gungor-ozkerim PS	Biomaterials Science	249
5	3D bioprinting for engineering complex tissues (2016)	Mandrycky C	Biotechnology Advances	247
6	Three-dimensional bioprinting of thick vascularized tissues (2016)	Kolesky DB	Proceedings of the National Academy of ScienceS of the United States of America	233
7	The bioink: A comprehensive review on bioprintable materials (2017)	Hospodiuk M	Biotechnology Advances	232
8	3D bioprinting of collagen to rebuild components of the human heart (2019)	Lee A	Science	212
9	Bioink properties before, during and after 3D bioprinting (2016)	Holzl K	Biofabrication	193
10	3D Bioprinting of Vascularized, Heterogeneous Cell-Laden Tissue Constructs (2014)	Kolesky DB	Advanced Mmaterials	189

**FIGURE 7:**
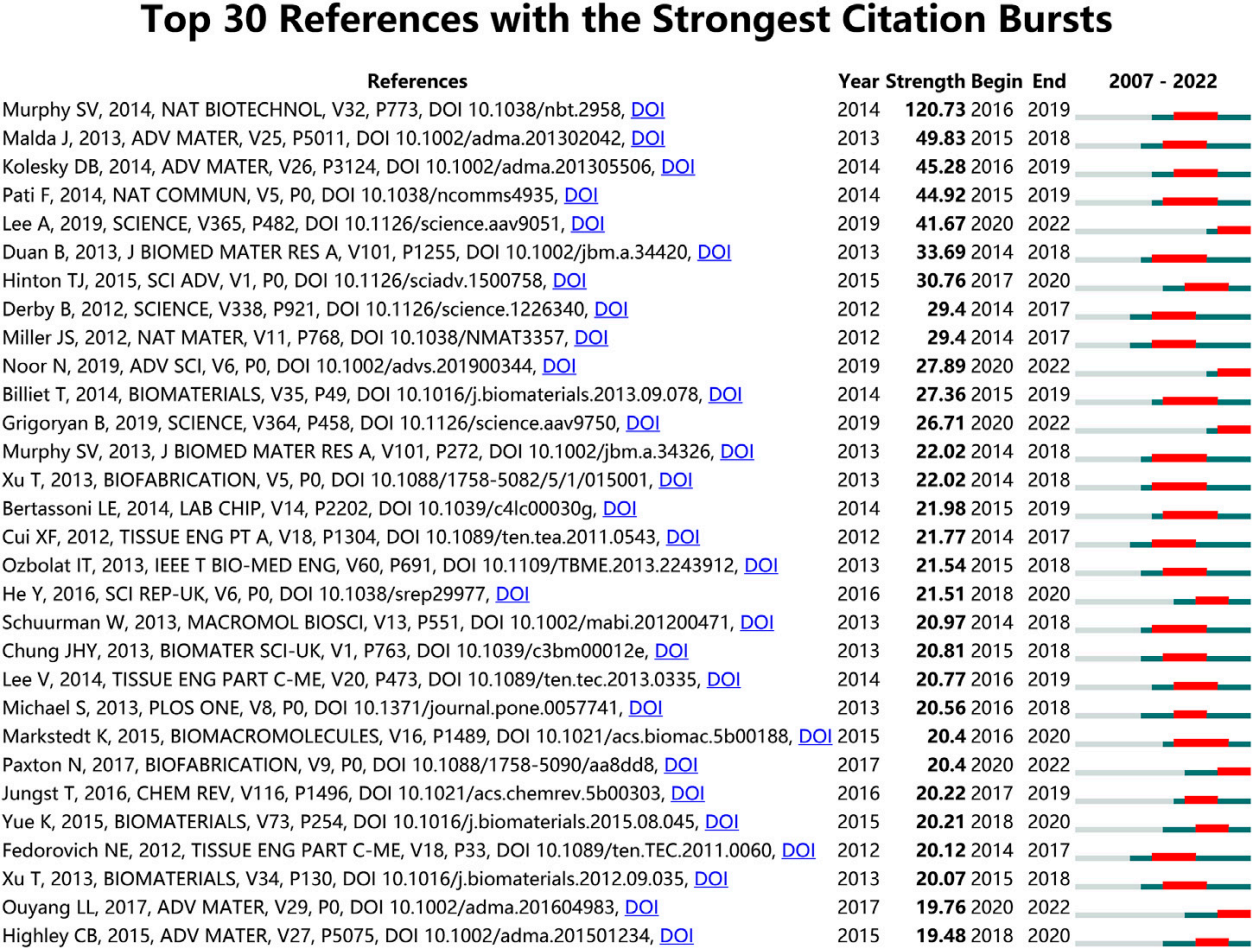
Top 30 references with the strongest citation bursts in 3D bioprinting research. Ranked by strength. The bars in red stands for a burst period for the references.

Analysis of the references is helpful to understand the development of a field. Figure 7 illustrates the top 30 references with the strongest citation bursts, all of which begun in 2014. Notably, the paper with the greatest number of co-citations (*n* = 470) and the highest strength of citation bursts (Strength = 120.73) was published in Nature Biotechnology by Murphy SV et al. in 2014 (Murphy and Atala, 2014). The second top co-cited publication was that by Kang HW et al. published in Nature Biotechnology (Kang et al. 2016). The third most co-cited paper was published in Biomaterials by Ozbolat and Hospodiuk (2016). Among all thirty articles, 6 are reviews and 24 are original research. In the top ten references with the strongest citation bursts, three of them overlap with the top 10 most co-cited references (Kolesky et al. 2014; Murphy and Atala, 2014; Lee et al. 2019), and two of the remaining seven articles are review papers mainly about engineering hydrogels for biofabrication (Malda et al. 2013), printing and prototyping of tissues and scaffolds (Derby, 2012). In the remaining five studies, Most of the studies are about different bioinks, including alginate/gelatin hydrogels (Duan et al. 2013), carbohydrate glass (Miller et al. 2012), decellularized extracellular matrix (Pati et al. 2014) and soft protein/polysaccharide hydrogels (Hinton et al. 2015). The development and improvement of bioinks are important components of 3D bioprinting research, as confirmed by early research directions. Besides, in addition to improving the biological ink matrix, research on cells is also very important. Noor et al. (2019) induced patient cells to become pluripotent stem cells and differentiated them into cardiac and endothelial cells. They used these two types of cells, along with collagenous nanofibers hydrogel, and 3D bioprinting technology to construct a cellularized human hearts with a natural architecture, this method demonstrated the potential for designing personalized tissues and organs.

**FIGURE 8:**
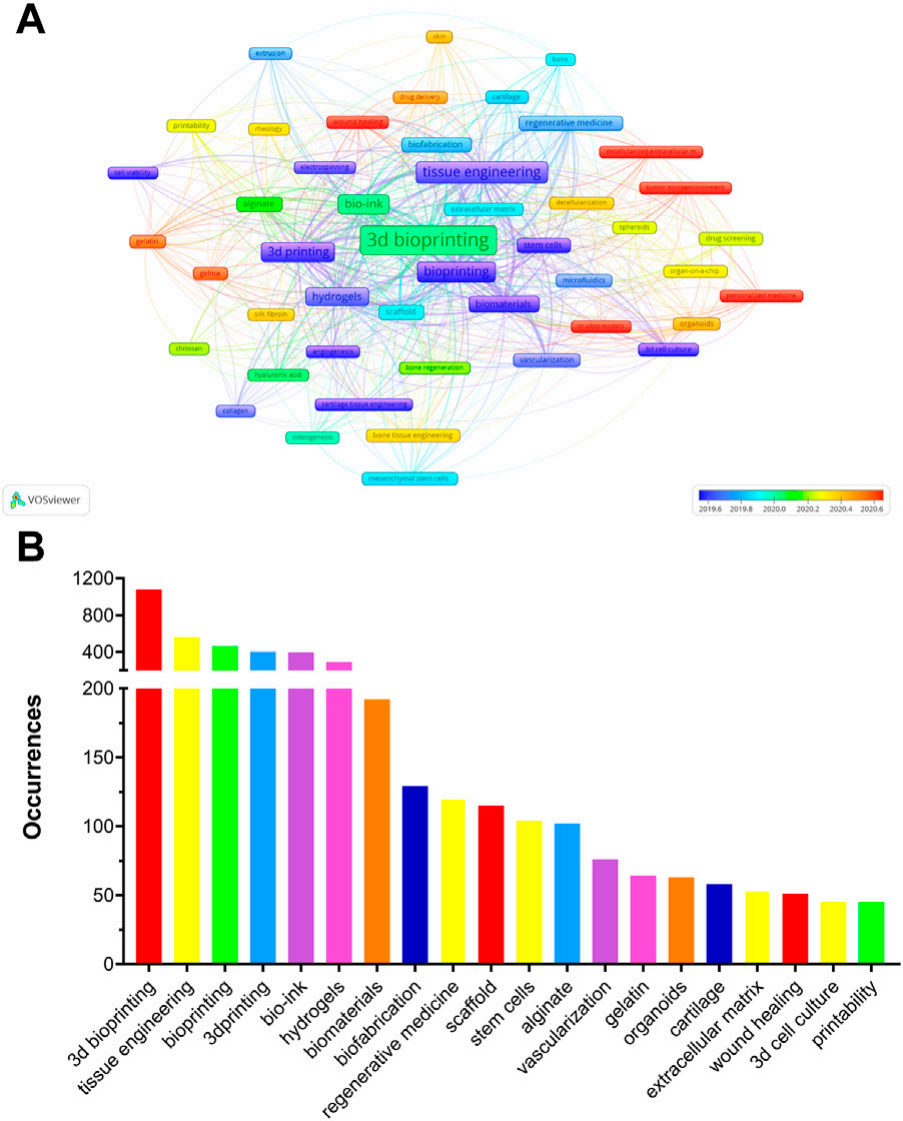
**(A)** The overlay of author keywords in 3D bioprinting research. The size of notes/words represent occurrences. The colour of the nodes indicated average publication year based on the strip of colour. **(B)** Top 20 author keywords with most frequent occurrences in 3D bioprinting research.

Analysis of Co-occurring Keywords and Related Genes; Keywords can further represent the hot issues in a related research field during a certain period, and recently erupted keywords can represent current research hotspots (Zhang et al. 2020; Wang H. et al. 2022). A total of 5,053 author keywords were identified, of which 49 with a minimum of 20 occurrences were extracted to create an overlay visualization map (Figure 8A). The top 20 author keywords based on the number of occurrences are shown in Figure 8B. 3D Bioprinting, Tissue Engineering, Bioprinting, 3D Printing, Bio-ink, Hydrogels, Biomaterials, Biofabrication, Regenerative Medicine, and Scaffold were ranked in the top ten in the occurrences of author keywords. As shown in Table 5, Cartilage Tissue Engineering, Cell Viability, Extrusion, Bioprinting, and Vascularization were the top five author keywords with the highest number of average citations. The top 30 author keywords of the average publication year are listed in Supplementary Table S2. Of these, “Decellularized Extracellular Matrix,” “*In Vitro* Models,” “Personalized Medicine,” “GelMA,” and “Wound Healing” were the top five author keywords with the newest average publication year. It is not surprised that keywords such as “3D bioprinting,” “Bio-ink,” “Tissue engineering” and “hydrogels,” et al. have become high-frequency keywords in recent times. Interesting to note is that according to the timeline, “Decellularized Extracellular Matrix,” “*In Vitro* Models,” “Personalized Medicine” and “Tumor microenvironment” have become the latest high-frequency keywords. Therefore, we speculate that these may be the hot research directions in the field of 3D bioprinting in the near future.

**TABLE 5:** The top 30 author keywords of average citations in 3D bioprinting research.

Rank	Author kewords	Average publication year	Occurrences	Link strength	Average citations
1	cartilage tissue engineering	2019.60	20	40	65.40
2	cell viability	2019.04	25	45	54.48
3	Extrusion	2019.78	27	72	50.19
4	bioprinting	2019.43	464	923	48.11
5	vascularization	2019.68	76	170	44.03
6	hydrogels	2019.66	290	668	44.01
7	drug screening	2020.23	40	79	43.95
8	stem cells	2019.37	104	236	43.72
9	Rheology	2020.32	25	53	40.32
10	biomaterials	2019.60	192	474	39.85
11	bone tissue engineering	2020.34	44	92	39.77
12	3d printing	2019.61	402	757	39.50
13	mesenchymal stem cells	2019.88	40	77	38.85
14	tissue engineering	2019.63	563	1,287	37.43
15	regenerative medicine	2019.77	120	327	37.07
16	bio-ink	2020.04	395	925	37.05
17	angiogenesis	2019.12	27	53	36.33
18	drug delivery	2020.46	36	68	33.14
19	3d cell culture	2019.51	45	98	32.67
20	printability	2020.25	45	112	31.56
21	Collagen	2019.68	34	67	31.18
22	extracellular matrix	2019.90	53	150	29.04
23	biofabrication	2019.81	129	291	28.74
24	electrospinning	2019.63	33	68	28.30
25	hyaluronic acid	2020.05	37	77	27.57
26	3d bioprinting	2020.04	1,081	1,541	26.72
27	Alginate	2020.12	102	231	25.72
28	decellularized extracellular matrix	2020.92	27	56	25.59
29	Gelma	2020.56	47	80	24.89
30	bone regeneration	2020.17	30	64	24.73

*Ranked based on average citations.

**FIGURE 9:**
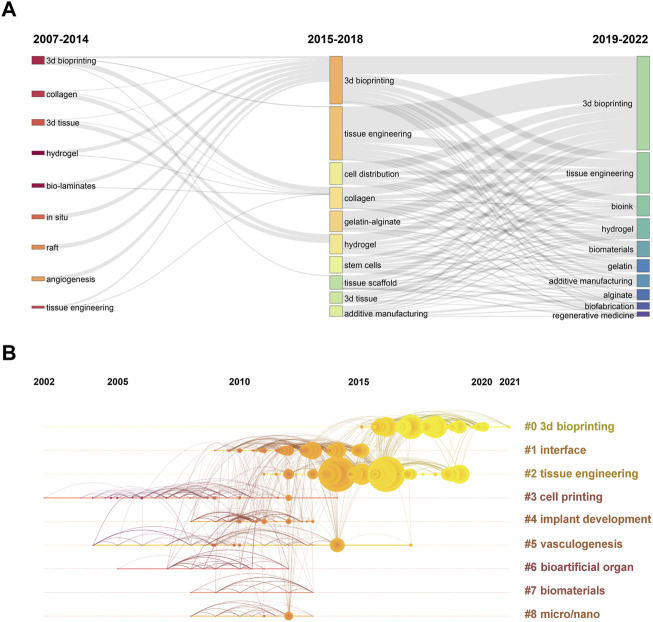
**(A)** Three-field plot of author keywords in 3D bioprinting research. The thick of the notes represent the relative co-occurrence frequency in a specific period. **(B)** The timeline view of co-cited reference in 3D bioprinting research. Years from 2002 to 2021 are arranged horizontally at the top, 9 clusters based on author keyword were identified and listed on the right. The larger the size of the circle, the more studies on the theme.

**FIGURE 10:**
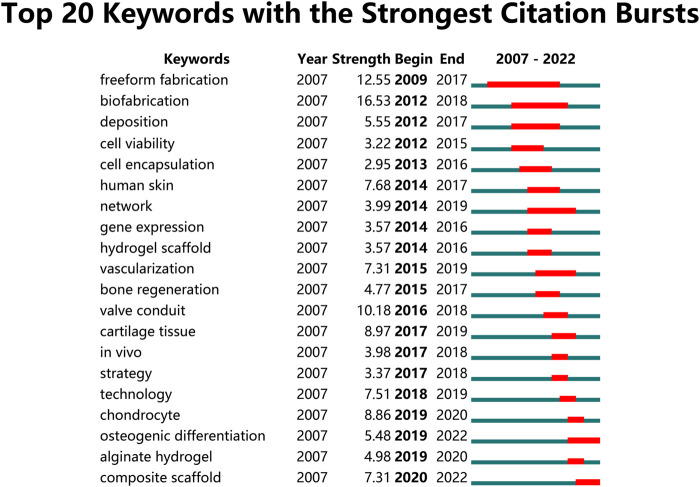
Top 20 author keywords with the strongest citation bursts in 3D bioprinting research. Ranked by begin year of citation burst. The bars in red stands for a burst period for the keywords.

Moreover, the results of thematic evolution analysis are shown in Figure 9A. The top 10 author keywords with the most occurrences in the corresponding period were identified. A three-field plot was applied to illustrate the evolution of three periods in the field of 3D bioprinting. As indicated in Figure 9B, the nodes indicate co-cited references in the timeline view map. All the co-cited references could be clustered into nine specific clusters based on the author keywords (Modularity Q value = 0.69, Weighted mean silhouette value = 0.86), including “#0 3D bioprinting,” “#1 interface,” “#2 tissue engineering,” “#3 cell printing,” “#4 implant development,” “#5 vasculogenesis,” “#6 bioartificial organ,” “#7 biomaterials,” and “#8 micro/nano.” Tissue engineering plays a very important role in the field of 3D bioprinting without doubt. “Vasculogenesis” is a new research direction in the field of 3D bioprinting in recent years. Due to the dependency of cells on oxygen and nutrients, vascularization during the 3D bioprinting process is a key focus for constructing engineered tissues and organs (Anthon and Valente, 2022). Moreover, the top 20 author keywords with the strongest citation bursts are shown in Figure 10. Freeform fabrication was once the hottest topic of interest in this research field, with the strongest citation burst. Chondrocyte, osteogenic differentiation, alginate hydrogel, and composite scaffold are four keywords with citation burst in the recent 4 years, which could represent the current research hotspots in this field. The weighted mean silhouette value was 0.86 and the modularity Q was 0.69, indicating the rationality of this clustering method.

## 4 Discussion

### 4.1 General information

The development of the annual output and the number of citations are useful indicators for the identification of trends in scientific research areas. By analysing the publication of global scientific publications, we can better understand the characteristics and current status of global scientific publications (Qin et al. 2020). The growing research fronts and future research interests in the field of 3D bioprinting over the past nearly 20 years are identified in this study. The data extracted from WoSCC database showed that 12,728 authors from 2,733 institutions in 83 countries/regions published 3,327 papers related to 3D bioprinting in 752 journals between 1 January 2007, and 30 November 2022.

Based on the trend line fitting the results shown in Figure 1, the numbers of published papers showed a rapid growth trend. The number of articles published each year did not exceed 100 before 2014, as the 3D bioprinting technology had just been proposed, while there were still many technical problems that had not been solved. With the development and commercialization of 3D bioprinters and bio-ink, an increasing number of research studies have been carried out in the field of 3D bioprinting, which explains the explosive growth of 3D bioprinting-related research after 2014 (Nishiyama et al. 2009; Guillemot et al. 2010; Chang et al. 2011; Mironov et al. 2011). Based on this growth trend, we believe that 3D bioprinting attracted more research attention and interest these years and predict that the number of papers in this field will further increase.

### 4.2 Countries and institutions

Based on the analyses of countries/regions and institutions shown in Figure 2, the United States and China were the two most productive countries. Furthermore, researchers from these two countries had more communication and cooperation in the 3D bioprinting field. This result was also found in 3D bioprinting-related fields, such as tissue engineering (Santisteban-Espejo et al. 2018), biomaterials in osteogenesis (Wang J. et al. 2022), nanocomposite hydrogels (Zhao et al. 2022) and stem cell precision medicine (Liu et al. 2022). It has been reported that research outputs are positively correlated with the gross domestic product (Tang et al. 2021c; Ding et al. 2022). Furthermore, China and the United States also spend the most research and development funds in this field, which can explain why the number of papers they publish is 1.3-fold higher than the total of any other country in the remainder top 10 countries. South Korea and Germany also had a number of publications exceeding 200, and researchers from the two countries also had close communication with the United States and China, suggesting that communication and cooperation is an important way to promote the development of this field.

Similar to the country distribution, most of the ten most prolific publishing institutions were in the United States and China. Harvard Medical School, Tsinghua University, Chinese Academy of Sciences, Zhejiang University, and Wake Forest School of Medicine, the best scientific research institutions in the United States and China, ranked in the top five in the list of the most prolific institutions. Obviously, excellent scientific research institutions are in a leading position in most fields and lead the way. University of California-Los Angeles has a top centrality of 0.18, suggesting that it may play an essential role in the 3D bioprinting field. International cooperation between different institutions is active as well, which is critical to the prosperity of this field (Feng et al. 2022).

### 4.3 Authors and Co-cited authors

Recognizing the influential scholars in a specific field can provide younger researchers with guidelines and direction. Among the authors with the most publications shown in Figure 4 and Table 2, Dr. Anthony Atala from Wake Forest School of Medicine was the leader with 63 articles and 113.14 times average citations per paper. As the director of the Wake Forest Institute for Regenerative Medicine, he and his team have developed 15 clinically-used technology-based applications, including muscle, urethra, cartilage, reproductive tissues, and skin. Furthermore, thay have developed specialized 3D printers to engineer tissues and achieved progress in organoids and body-on-a-chip systems (Atala et al. 2012; De Filippo et al. 2015; Kang et al. 2016; Wang et al. 2018; Jorgensen et al. 2020; Murphy et al. 2020). As the world’s leading expert in 3D bioprinting, Dr. Anthony has created many “world first” records in the 3D bioprinting field, such as developing a 3D bioprinter (the Integrated Tissue and Organ Printing System) to print living tissue structures over 14 years (Kang et al. 2016), and a novel hybrid strategy based on 3D bioprinting to fabricate endothelialized myocardium (Zhang et al. 2016). Another researcher is equally noteworthy, Dr. Ali Khademhosseini from the department of bioengineering, University of California-Los Angeles and Terasaki Institute for Biomedical Innovation, who has 35 articles published but 122.97 times average citations per paper and 27 H-index. Dr. Khademhosseini has made great contributions in the field of 3D bioprinting, including perfusable vascular constructs using direct 3D bioprinting (Jia et al. 2016), oxygenated cell-laden gelatin methacryloyl constructs using 3D bioprinting (Erdem et al. 2020) and a gelatin methacryloyl (GelMA)-based bio-ink for 3D bioprinting (Adib et al. 2020). Certainly, Dr. Khademhosseini and his team have made great progress in applying bioengineering solutions to precision medicine. Notably, all the top 10 authors have achievements each year, therefore, we infer that the 3D bioprinting field has excellent prospects. The collaboration among the top 25 highly productive authors is not as extensive as expected, which may be due to their different countries and research institutions or the impact of the COVID-19, et al.

As for the co-cited authors shown in Figure 5 and Table 2, Dr. Sean V Murphy from Wake Forest Institute for Regenerative Medicine and Dr. Ibrahim T Ozbolat from the Department of Engineering Science and Mechanics, Penn State University, have published several highly cited review papers on 3D bioprinting (Ozbolat and Yu, 2013; Dababneh and Ozbolat, 2014; Murphy and Atala, 2014; Ozbolat and Hospodiuk, 2016; Hospodiuk et al. 2017). These review articles help other scholars quickly and accurately understand the field of 3D bioprinting. In short, the top 10 authors with the most influential publications and the most co-citations have been leading the entire discipline forward.

### 4.4 Influential journals

Journals are an important vector for the dissemination of academic research results. We summarized the co-citation visualization network of the most influential journals in the field of 3D bioprinting, making it easier for researchers to choose the most suitable journals to submit papers (Cheng et al. 2022). As shown in Figure 6 and Table 3, Tissue Engineering Part A, Biofabrication, Advanced Healthcare Materials, International Journal of Bioprinting, and Frontiers in Bioengineering and Biotechnology take up the top five positions. Tissue Engineering Part A published the most 3D bioprinting research. This is an authority journal in tissue engineering that mainly focuses on the fundamental and applied experimental aspects for the development of therapeutic strategies to repair or regenerate tissue and organ function. It is worth noting that Frontiers in Bioengineering and Biotechnology is the journal mainly focusing on bioengineering and biotechnology. We believe that this journal will occupy a more important position in the field of 3D bioprinting in the future. Meanwhile, journals in JCR Q1 division accounted for 70% of the top 10 journals, indicating that these journals attract the interest of many researchers and play essential roles in the 3D bioprinting research field.

The dual-map overlay of journals stands for the topic distribution of academic journals (Chen and Leydesdorff, 2014). Figure 6 shows four citation paths, Physics/Materials/Chemistry co-cited journals to Chemistry/Materials/Physics and Molecular/Biology/Genetics, Molecular/Biology/immunology co-cited journals to Chemistry/Materials/Physics and Molecular/Biology/Genetics. It represents the theme distribution of the corresponding journal. The citing journals are on the left and the cited journals on the right. The saffron and purple paths reflect the relationships between the citing and cited journals, respectively (Zhang et al. 2021a). This result indicates that 3D bioprinting is an important interdisciplinary field, and that the cooperation between Chemistry/Materials/Physics and Molecular/Biology/Genetics/immunology is the current mainstream research direction.

### 4.5 The top 10 most Co-cited references

A co-cited reference means that an article was cited as a reference in different articles (Shen et al. 2022). The knowledge base for this study is a compilation of references cited by the included papers. Therefore, it is not the same as a highly cited paper.

According to the references listed in Table 4, most papers (60%) are reviews. Nature Biotechnology published the most co-cited reference papers by Dr. Sean V Murphy and Dr. Anthony Atala in 2014 with a total of 470 co-cited times until now (Murphy and Atala, 2014). This review article provides a comprehensive review of the applications of 3D bioprinting in tissue and organ engineering, mainly focusing on the strategy for printing organizational structure, different types of bioprinters and their impact on printing tissue structures, the process of printing tissue, limitations of current techniques, and challenges for future research. Certainly, the two top researchers initiated the comprehensive development of 3D bioprinting with this article.

Two years later, Dr. Anthony Atala and his team published the second most co-cited publication in the same journal (Kang et al. 2016). The team presented an integrated tissue–organ printer that can fabricate stable, human-scale tissue constructs of any shape. They constructed mandible bone, calvarial bone, cartilage and skeletal muscle. This innovative 3D bioprinter that can produce human-scale tissue is the prototype of the most commercial 3D bioprinters.

The third most co-cited paper was a review article by Dr. Ibrahim T Ozbolat and Dr. Monika Hospodiuk in 2016 in Biomaterials (Ozbolat and Hospodiuk, 2016). Extrusion-based bioprinting (EBB) has made noteworthy progress from 2006 to 2016, and this article comprehensively reviewed this technology for the first time. Specifically, the authors discussed the current progress in EBB technology, and highlighted the future directions for upgrading the technology to produce applicable products for tissue engineering and regenerative medicine. The rest of the top 10 most co-cited articles were published between 2014 and 2019 (Table 4).

### 4.6 The top references with the strongest citation burst

References with high burst values in a particular time period indicate that they have received a lot of attention during the corresponding time span (Sun et al. 2022). The references with the top 30 strongest citation bursts are listed in Figure 7. As expected, the strongest citation burst reference was still the review article published by Dr. Sean V Murphy and Dr. Anthony Atala in 2014 in Nature Biotechnology with a 120.73 strength, proving once again the importance of this article in the field (Murphy and Atala, 2014). Dr. Jos Malda, et al. published a 25th anniversary article about engineering hydrogels for biofabrication in Advanced Materials in 2013 (Malda et al. 2013). As the second most cited burst reference, this article mainly focused on the important physicochemical aspects for the development and characterization of hydrogels for biofabrication, and discussed how they may impact the composition and properties of hydrogel bio-inks in the future.

The time of citation burst of all these 30 references started after 2014 and ended approximately in 2022, further proving that the 3D bioprinting technology-related research achieved rapid development after 2014 and became a hotspot in tissue engineering and regenerative medicine recently.

### 4.7 The hot topics and future trends of 3D bioprinting

Analysis of author keyword occurrences can reveal the research interest areas and hotspots in a specific field (Xiao et al. 2017). The author keywords with high occurrence are shown in Figure 8. 3D Bioprinting, Tissue Engineering, Bioprinting, Bio-ink, 3D Printing, Hydrogels, Biomaterials, Biofibrication, Regeneration Medicine, and Scaffold were listed in the top ten. These can be regarded as the hotspot research interest areas in 3D bioprinting. Besides, Cartilage Tissue Engineering, Cell Viability, Extrusion, Bioprinting and Vascularization have the top five highest average citations in Table 5.

Furthermore, the changing of research hotspots and directions are capable of predicting the future trends in the field with ease as shown in Figure 9A. When comparing the changes in research hotspots between the past 8 years and the recent 4 years, we can clearly see that there have been significant changes. Firstly, 3D bioprinting has gained increasing attention, while the hotspot of tissue engineering has slightly decreased. However, since 3D bioprinting is a branch of tissue engineering research, we can understand this shift in hot topics as 3D bioprinting gaining more attention in the field of tissue engineering. In addition, it is not surprising that bioink has emerged as a hot keyword in the past 4 years. As one of the most crucial components of 3D bioprinting technology, it is probably one of the hot trends for future research. The attention given to hydrogels has significantly increased, and as a material that is, highly suitable for bioink applications, it is widely recognized as a future development trend in 3D bioprinting. Biomaterials have also emerged as a hot keyword in the past 4 years, and we believe that the application of 3D bioprinting in biomaterials production will experience significant development in the coming years. Gelatin and alginate are two of the earliest discovered hydrogels, but their attention has decreased in the past 4 years as various other hydrogels with more advantages have been developed. It is undeniable that they are high-quality hydrogels and have been applied in various fields, but their future research will gradually decrease. Besides, as a part of the additive manufacturing and biofabrication field, the development of 3D bioprinting has promoted the advancement of additive manufacturing and biofabrication technology. Regenerative medicine, as the biggest application direction of 3D bioprinting, is probably a highly focused field in the future.

Analysis author keywords of the average publication year can also make certain predictions about the future development trends in the field as shown in Supplementary Table S2, Decellularized Extracellular Matrix, *In Vitro* Models, Personalized Medicine, Gelatin Methacryloyl, and Wound Healing are the top five latest author keywords.

Based on the bibliometric analysis, we make the prediction that bioink, hydrogels, tissue engineering, decellularized extracellular matrix (DECM), *in vitro* models, personalized medicine, regenerative medicine, GelMA, cell viability and vascularization will be the hot trends of 3D bioprinting in the present and nearly future. The following is our further analysis.

#### 4.7.1 Bio-ink and hydrogels

Bio-ink was defined as materials which are capable to include cells and other bioactive components for the use in biofabrication (Heid and Boccaccini, 2020). Bio-inks cannot be confused with biomaterial-inks, which have always been considered the materials used in biofabrication. Bio-ink is a material that must be first printed, sterilized, and seeded with cells to enable scaffolding components or generate hybrid supports to improve the mechanical resistance of 3D printed specimens (Groll et al. 2018). Bio-inks always consist of cells and scaffolding material before printing, meaning that the cells are already evenly distributed in the scaffold during the printing process (Tiwari et al. 2021). The bio-inks must have the ability: i) to act as a platform for cells to adhere, grow, spread and proliferate; ii) provide adequate structural support during and after printing; and iii) protect cells from printing stress damage (Chimene et al. 2020; Zhang et al. 2021b). To further analyse the recent progress in bio-ink, we conducted a search using “bioink” OR “bio-ink” OR “bioinks” OR “bio-inks” as the keywords in the WOS core collection database. We obtained 1810 related articles (original research and reviews) and extracted all author keywords, then screened for all keywords related to bio-ink materials. Based on the analysis, we constructed a time-based co-occurrence network of the keywords (Supplementary Figure S2). As shown in Supplementary Figure S2, there have been significant advancements in bio-inks materials in recent years, with more and more materials being discovered for use in bioprinting. Before 2015, only a few types of bioinks were available for 3D bioprinting, including combined biopolymers and composite materials (Pataky et al. 2012; Malda et al. 2013; Wüst et al. 2014; Das et al. 2015; Kesti et al. 2015), fibrin (Cui and Boland, 2009; Cubo et al. 2016), oxidized alginate (Jia et al. 2014), and spherical cell aggregates (Jakab et al. 2004; Levato et al. 2014). From 2015 to 2018, a large number of new materials that can be used as bioinks have emerged. These include, but are not limited to, alginate (Axpe and Oyen, 2016), hyaluronic acid (Lee et al. 2018; Petta et al. 2018; Weis et al. 2018), collagen (Diamantides et al. 2017; Włodarczyk-Biegun and Del Campo, 2017), decellularized extracellular matrix (dECM) (Pati et al. 2014; Kim et al. 2017; Choudhury et al. 2018; Nam and Park, 2018), gelatin methacryloyl (GelMA) (Yue et al. 2015; Van Hoorick et al. 2019), silk fibroin (Chawla et al. 2018), spider silk (DeSimone et al. 2017) and agarose (Duarte Campos et al. 2015; Forget et al. 2017; López-Marcial et al. 2018). Besides, it is worth noting that gelatin is mostly used in combination with other materials in bioinks, and its main function is to maintain the shape of the printing scaffold before ink crosslinking. After 2018, more materials have been discovered to be suitable for use as bio-inks., including chitosan (Kołodziejska et al. 2021; Xu J. et al. 2022; Lazaridou et al. 2022), gellan gum (Cernencu and Ioniță, 2023), hydroxyapatite (Heid and Boccaccini, 2020), pectin (Mahendiran et al. 2021; Merli et al. 2022), cellulose (Ahlfeld et al. 2020; Zennifer et al. 2021) and polysaccharides (Naranda et al. 2021; Teixeira et al. 2022). Meanwhile, we also noticed that dECM, GelMA, and alginate have received more attention recently. In addition, more studies are combining two or three existing bio-inks to obtain a better bio-ink through complementary advantages and disadvantages, which is also one of the hot research directions in this field.

Based on the material requirements, hydrogels are gradually being used in bio-inks. Hydrogel biomaterials include alginate, gelatin, collagen, fibrin/fibrinogen, gellan gum, hyaluronic acid, agarose, chitosan, silk, decellularized extracellular matrix (dECM), poly (ethylene glycol), and Pluronic. Hydrogels have many attractive features for use as bio-inks. As they are biocompatible and typically biodegradable, most of them are easy for cells to adhere, grow, spread and proliferate on (Gungor-Ozkerim et al. 2018). Furthermore, different cross-linking methods enable hydrogels to be applied to various cells and tissues (Basu et al. 2016; Hospodiuk et al. 2017; Paxton et al. 2017; Vázquez-González and Willner, 2020; Xu et al. 2020). Although the abundance of hydrogels offers great potential for tissue engineering, their applications in 3D bioprinting are still limited due to the lack of bioprinting capabilities (Hospodiuk et al. 2017). Hydrogels do not contain specific proteins from the extracellular matrix of cells, so it is hard to simulate the “native environments” for specific cells. Furthermore, the physical properties of hydrogels may affect the normal biological function of cells. Then, the degradation of hydrogels and strength of scaffold should also be noticed (Hospodiuk et al. 2017; Osidak et al. 2020; Petta et al. 2020). Not surprisingly, bio-inks and hydrogels have been the most studied topic in 3D bioprinting. With the development of regenerative medicine and tissue engineering, research on new hydrogel materials with better material properties might become a research hotspot in the future.

#### 4.7.2 Tissue engineering

Tissue engineering is one of the hottest research fields in recent years, and it has been widely applied to musculoskeletal tissue, oral tissue, cardiovascular tissue, urogenital tissue, ocular tissue, and so on (Gupta and Bit, 2022). Based on its important role in 3D bioprinting, we conducted a brief bibliometric analysis of tissue engineering research in the past 4 years. A total of 30,738 documents (article and review) and 39,849 author keywords were analyzed, and Frontiers in Bioengineering and Biotechnology has published the most research (785 articles). As shown in Supplementary Figure S3, we obtained the top 60 author keywords in tissue engineering research over the past 4 years and they were further analyzed by VOSviewer to create the density visualization map (Supplementary Figure S3A) and all the sixty keywords were subjected to cluster analysis, where the size of the node representing each keyword corresponds to its frequency of occurrence (Supplementary Figure S3B). We found that bone tissue engineering and cartilage tissue engineering were the most researched directions. Meanwhile, 3D bioprinting is also one of the hot research directions in tissue engineering, which is consistent with our previous analysis. From this, we can make further inference that the application of 3D bioprinting technology in bone and cartilage tissue engineering will continue to be a research hotspot in these two fields for some time to come, and technological advancements will bring more advantageous solutions for the repair of bone and cartilage injuries.

#### 4.7.3 Extrusion and bioprinting

There are four major 3D bioprinting methods, namely, extrusion, inkjet, stereolithography, and laser-assisted printing (Gillispie et al. 2020). EBB extrudes or dispenses continuous strands or fibers of biomaterials to form 3D scaffold structures, and is the first of the demonstrated bioprinting modalities and the most widely used 3D bioprinting technology (Landers et al. 2002; Pati et al. 2015; Zhang et al. 2021c). EBB has plenty of advantages compared to other 3D printing methods: 1) a variety of biomaterials and cell types can be used (Pati et al. 2014); 2) the scaffold can be constructed layer by layer with appropriate physiological cell density according to needs (Mironov et al. 2009a); 3) it is less damaging to cells in the construction process (Skoog et al. 2014; Ozbolat and Hospodiuk, 2016); 4) has great potential for stem cell growth, differentiation, and function (Chen, 2019). However, EBB still has some limitations. First, the commonly used bio-inks are limited by the inability to obtain high cell densities comparable to those found in native tissues (Zhang et al. 2021c), while scaffold-free multicellular spheroids with extrusion bioprinting and single-cell-only bioprinting techniques may be able to solve this problem (Mironov et al. 2009b; Norotte et al. 2009; Yu et al. 2016; Jeon et al. 2019). Second, as the resolution and throughput limits the development of EBB technology, one has to sacrifice one of these two contradicting functional parameters. We believe this problem can be solved by microfluidics technology, airflow motor control, and a microvascular multi-nozzle printhead (Hansen et al. 2013; du Chatinier et al. 2021; Tang G. et al. 2021). Therefore, we infer that developing 3D bioprinting methods and technologies with more advantages may become one of the hot trends in future research.

#### 4.7.4 DECM

DECM scaffold refers to biomaterials formed by human or animal organs/tissues with the removal of immunogenic cellular components using decellularized technologies (Hinderer et al. 2016). A dECM scaffold is mainly composed of extracellular matrix, which contains collagen, elastin, fibronectin, laminin, and matricellular proteins (Chen and Liu, 2016; Zhang et al. 2022). As dECM scaffolds maintain the physicochemical signals and biological performance after decellularization as well as provide mechanical support, they have been widely used in tissue engineering (Hoshiba, 2019; Kim et al. 2020). Despite this fact, not all dECM are suitable for 3D bioprinting; bioprintability, cell viability, mechanical and structural properties, as well as remodeling capability must be considered (Kim et al. 2020). The dECM still has lots of limitations, such as immunogenic properties, pro-remodeling response in host tissue, cellular cytoxicity from UV light and chemical agents in the process of cross-linking, and the integration of the cell-printed constructs in the body (Yeo et al. 2007; Wang et al. 2017; Schmitt et al. 2021). In general, the dECM scaffold has great prospects in 3D bioprinting, but several problems remain to be solved before practical application in the future.

#### 4.7.5 *In Vitro* Models

*In vitro* models are important tools to study the occurrence and development of diseases (Kaur et al. 2022). Many disease models have been created using 3D bioprinting, such as tumor models (Neufeld et al. 2022), osteochondral unit models (Santos-Beato et al. 2022), multicellular cardiac fibrosis models (Picchio et al. 2022), as well as liver (Guagliano et al. 2022) and skin models (Phang et al. 2022). In recent years, organoid technology has rapidly developed as an *in vitro* model. Organoids are derived from pluripotent stem cells or isolated organ progenitors that differentiate to form an organ-like tissue exhibiting multiple cell types that self-organize to form a structure not unlike the organ *in vivo* (Lancaster and Knoblich, 2014; Rossi et al. 2018). Organoid technology is further developed because of the 3D bioprinting technology, allowing the distribution of cells in organoids to be adjusted as needed and better organ simulation (Chakraborty et al. 2022). Therefore, we believe that the combinatorial perspectives of tissue engineering, organoids, and 3D bioprinting are promising.

#### 4.7.6 GelMA

GelMA is a hydrogel synthesized by the chemical reaction between the hydroxyl and amine groups of the amino acid residues and methacrylic anhydride (Van Den Bulcke et al. 2000). As GelMA exhibits great compatibility with various cells, biodegradability, and accessibility (Loessner et al. 2016), it is widely used in hard and soft tissue engineering (Li et al. 2018; Chimene et al. 2020; Unagolla and Jayasuriya, 2020; Rajabi et al. 2021). Besides, due to its similarity to the main component in the extracellular matrix, GelMA has been used in drug and gene delivery, as well as in wound healing applications (Yue et al. 2015; Ayoub et al. 2022; Hölzl et al. 2022; Moniruzzaman et al. 2022; Tozar et al. 2022). As one of the best 3D bioprinting materials, GelMA still has some limitations, such as low viscosity at room or higher temperature and rapid degradation in body tissue (Seyedmahmoud et al. 2019; Rajabi et al. 2021). Nonetheless, GelMA has very good prospects in 3D bioprinting.

#### 4.7.7 Personalized and regenerative medicine

3D bioprinting has played a significant role in personalized medicine, especially in the field of cancer research. In the treatment of tumors, the most appropriate treatment plan should be adopted for different patients, and 3D bioprinting technology can achieve personalized tumor organoid and simulate the tumor microenvironment to the greatest extent. Studying the sensitivity of drugs in this personalized organoid and designing the optimal treatment plan is the best solution pursued in clinical treatment, while also reducing the use of animal models (Jung et al. 2022). In addition, 3D bioprinting technology also has great potential in drug screening. Studying the efficacy of new drugs by constructing different tissue organoids and disease models is one of the hot research directions. Meanwhile, utilizing 3D bioprinting technology to construct personalized tissue organs and apply them in transplant medicine also can be one of the development directions for the future of personalized medicine (Ma et al. 2018).

Regenerative medicine is an important discipline focused on the regeneration of complex tissue and organ systems (Tang et al. 2019). Currently, 3D bioprinting has achieved results in the regenerative medicine research of various organs, including skin tissue, heart tissue, bone tissue, cartilage tissue, liver tissue, lung tissue, nervous tissue, and pancreatic tissue, among others (Matai et al. 2020). However, so far, 3D bioprinting technology still has many limitations in the field of regenerative medicine, including the insolubility, stability, and promotion of cell growth of bioinks (Ravnic et al. 2017). Therefore, the application of 3D bioprinting technology in the field of regenerative medicine also might be a hot research direction for the future.

#### 4.7.8 Cell viability and vascularization

As cells are subjected to various types of pressure during the 3D bioprinting process, cell viability is one of the most challenging issues (Kačarević et al. 2018; Xu H-Q. et al. 2022). The pressure generated during the 3D bioprinting process can affect cell signaling pathways and protein expression, which further impacts cell viability. Current methods mainly involve controlling pressure changes during the printing process, improving the shape and performance of the nozzle, adjusting overall parameters, and optimizing bioinks, among other techniques (Chang et al. 2008; Busch et al. 2015; Boularaoui et al. 2020). Therefore, improving cell viability as much as possible during the 3D bioprinting process is an urgent and important issue that needs to be addressed in this field of research in the near future.

Studies have shown that engineered tissues with a thickness greater than 1 mm have difficulty maintaining their normal cell viability without vascularization (Visconti et al. 2010; Laschke and Menger, 2012). Therefore, achieving vascularization in 3D bioprinting is crucial. Although there are currently several methods available for vascularization, including improved printing techniques, the use of bioinks that promote vascularization, and others, there is still a significant gap compared to normal tissue (Kong and Wang, 2023). Therefore, we believe that developing improved 3D bioprinting techniques for vascularization may be one of the future hotspots.

### 4.8 Limitations

All the bibliometric analysis articles have several inherent limitations and this study is no exception. First, all the data in this study were extracted from the WoSCC only. Although this database has been widely used in bibliometrics, papers only published in other databases were excluded from this study. Then, part of the data was analysed using software based on the strength of machine learning, which may lead to bias in data analysis. Additionally, Due to the time required for the publication of an article, new relevant literature may be published during this period, this is an unavoidable issue in current bibliometric research. Nonetheless, our research still provides a comprehensive overview of different directions in the field of 3D bioprinting and offers researchers more objective data, knowledge, and insight.

## 5 Conclusion

Using information visualization technology, this study fully summarizes the research progress, hotspots, and frontiers in the 3D bioprinting field. Exciting findings can provide a foundation for further research in this field and inspire the collaboration among potential partners and institutions. Research on 3D bioprinting has greatly developed and still has huge prospect in the future. The United States and China have absolute advantages in this field, and interested researchers may find cooperation opportunities in Harvard Medical School, Tsinghua University, Chinese Academy of Sciences, University of California-Los Angeles, and Wake Forest School of Medicine. Tissue Engineering Part A published the most articles in this field, and Dr. Anthony Atala was the most productive author. The academic exchange and cooperation between countries, institutions, and authors has promoted the development of this research field. Bio-inks and scaffolds have become a hotspot recently. The development of different hydrogels (especially GelMA) and dECM is expected to become an attractive direction in the next years. In addition, further studies on the modification of the EBB technique are key to promoting the progress of 3D bioprinting. Notably, the application of 3D bioprinting, especially in tissue engineering and *in vitro* models (organoids in particular), is the research Frontier in this field, and it is currently in an explosive period. Last but not least, the application of 3D bioprinting in personalized and regenerative medicine will continue to develop in the future, but the important issues of cell viability and vascularization need to be addressed first.

## Data Availability

The original contributions presented in the study are included in the article/Supplementary Material, further inquiries can be directed to the corresponding authors.
