# Supplementary material for: Global hotspots and emerging trends in 3D bioprinting research
Source: Front Bioeng Biotechnol. 2023 May 25;11:1169893. doi: 10.3389/fbioe.2023.1169893 (PMC10248473; doi:10.3389/fbioe.2023.1169893)
Supplement: Supplementary file 1 [file Table1.docx]

Supplementary Table 1. The top 30 author keywords of average publication year in 3D bioprinting research

| **Rank** | **Author kewords** | **Average publication year** | **Occurrences** | **Link strength** | **Average citations** |
| --- | --- | --- | --- | --- | --- |
| 1 | decellularized extracellular matrix | 2020.92 | 27 | 56 | 25.59 |
| 2 | in vitro models | 2020.92 | 24 | 47 | 13.88 |
| 3 | personalized medicine | 2020.89 | 20 | 51 | 11.00 |
| 4 | wound healing | 2020.71 | 51 | 102 | 22.61 |
| 5 | tumor microenvironment | 2020.64 | 25 | 54 | 18.84 |
| 6 | gelma | 2020.56 | 47 | 80 | 24.89 |
| 7 | gelatin | 2020.53 | 64 | 167 | 24.58 |
| 8 | drug delivery | 2020.46 | 36 | 68 | 33.14 |
| 9 | organoids | 2020.41 | 63 | 137 | 22.29 |
| 10 | skin | 2020.40 | 22 | 55 | 22.68 |
| 11 | decellularization | 2020.36 | 23 | 67 | 21.96 |
| 12 | silk fibroin | 2020.36 | 22 | 48 | 24.09 |
| 13 | bone tissue engineering | 2020.34 | 44 | 92 | 39.77 |
| 14 | rheology | 2020.32 | 25 | 53 | 40.32 |
| 15 | organ-on-a-chip | 2020.30 | 29 | 65 | 21.10 |
| 16 | printability | 2020.25 | 45 | 112 | 31.56 |
| 17 | spheroids | 2020.24 | 38 | 80 | 16.18 |
| 18 | drug screening | 2020.23 | 40 | 79 | 43.95 |
| 19 | chitosan | 2020.19 | 31 | 61 | 20.06 |
| 20 | bone regeneration | 2020.17 | 30 | 64 | 24.73 |
| 21 | alginate | 2020.12 | 102 | 231 | 25.72 |
| 22 | hyaluronic acid | 2020.05 | 37 | 77 | 27.57 |
| 23 | 3d bioprinting | 2020.04 | 1081 | 1541 | 26.72 |
| 24 | bio-ink | 2020.04 | 395 | 925 | 37.05 |
| 25 | osteogenesis | 2020.00 | 22 | 43 | 19.27 |
| 26 | scaffold | 2019.92 | 115 | 267 | 21.98 |
| 27 | extracellular matrix | 2019.90 | 53 | 150 | 29.04 |
| 28 | bone | 2019.90 | 30 | 87 | 22.03 |
| 29 | mesenchymal stem cells | 2019.88 | 40 | 77 | 38.85 |
| 30 | cartilage | 2019.86 | 58 | 148 | 24.67 |

*Ranked based on average publication year
