# Supplementary figures and images for: Global hotspots and emerging trends in 3D bioprinting research
Source: Front Bioeng Biotechnol. 2023 May 25;11:1169893. doi: 10.3389/fbioe.2023.1169893 (PMC10248473; doi:10.3389/fbioe.2023.1169893)

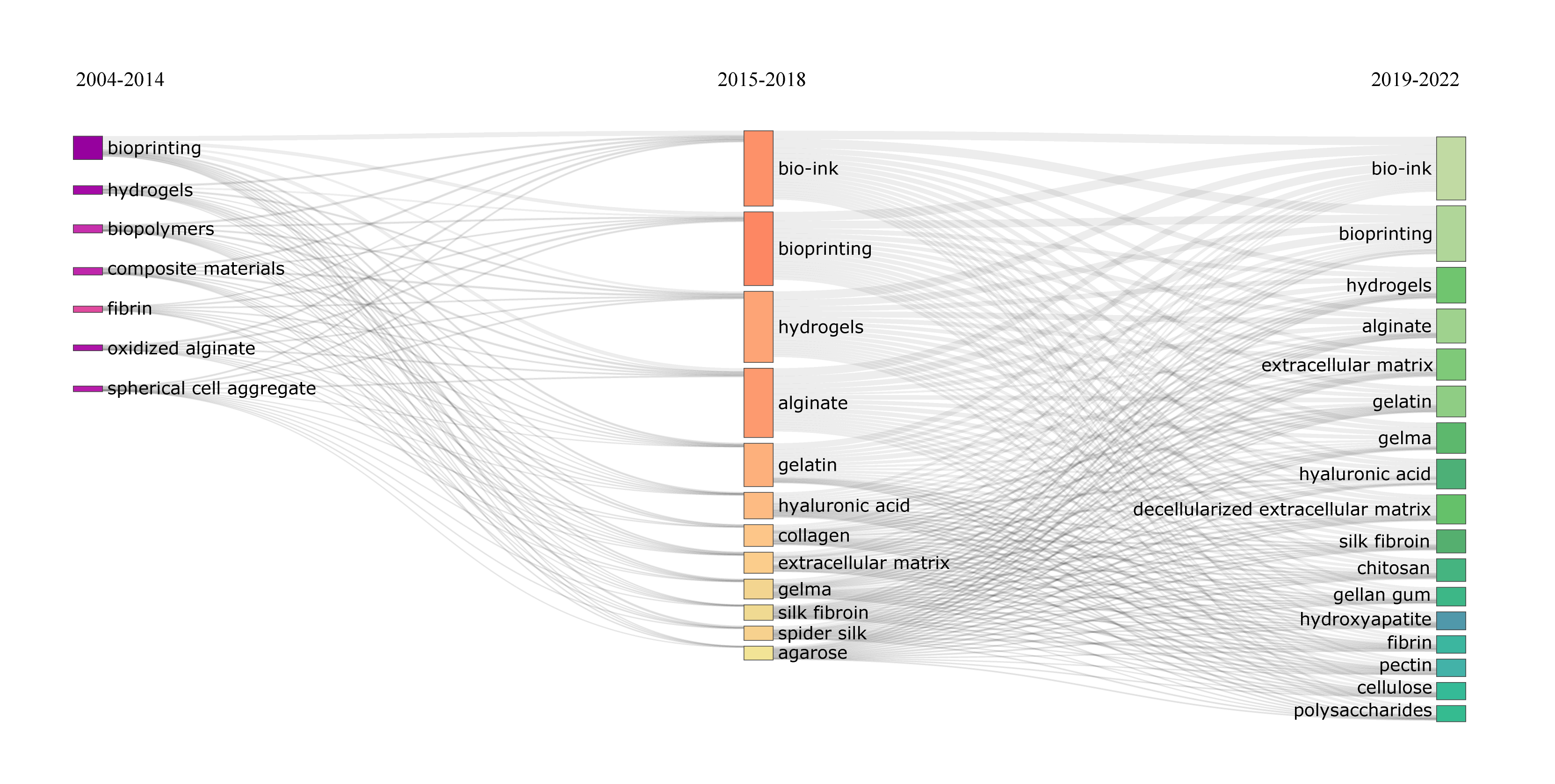

Supplement: Supplementary file 2 [file Image2.TIF]

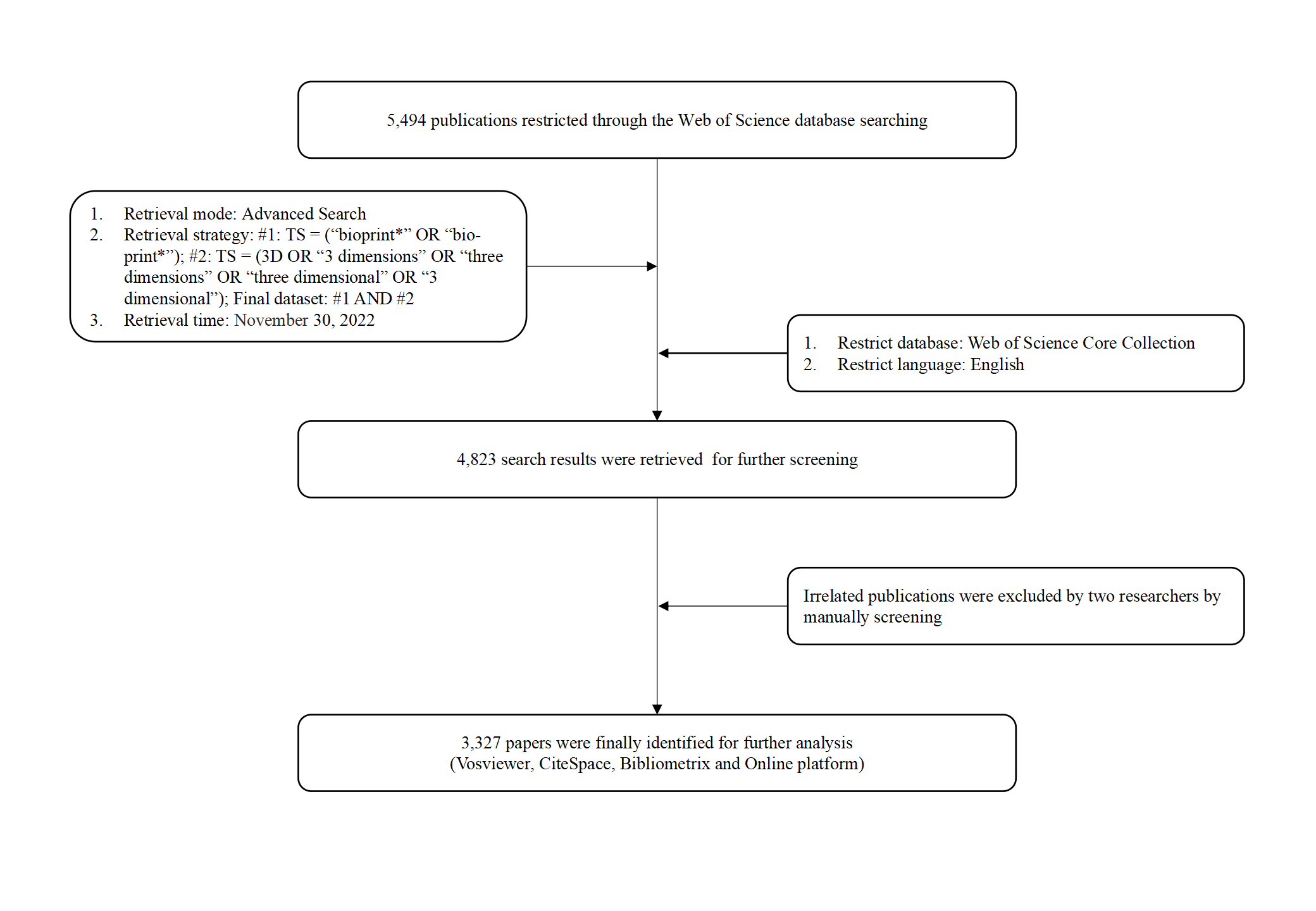

Supplement: Supplementary file 3 [file Image1.TIF]

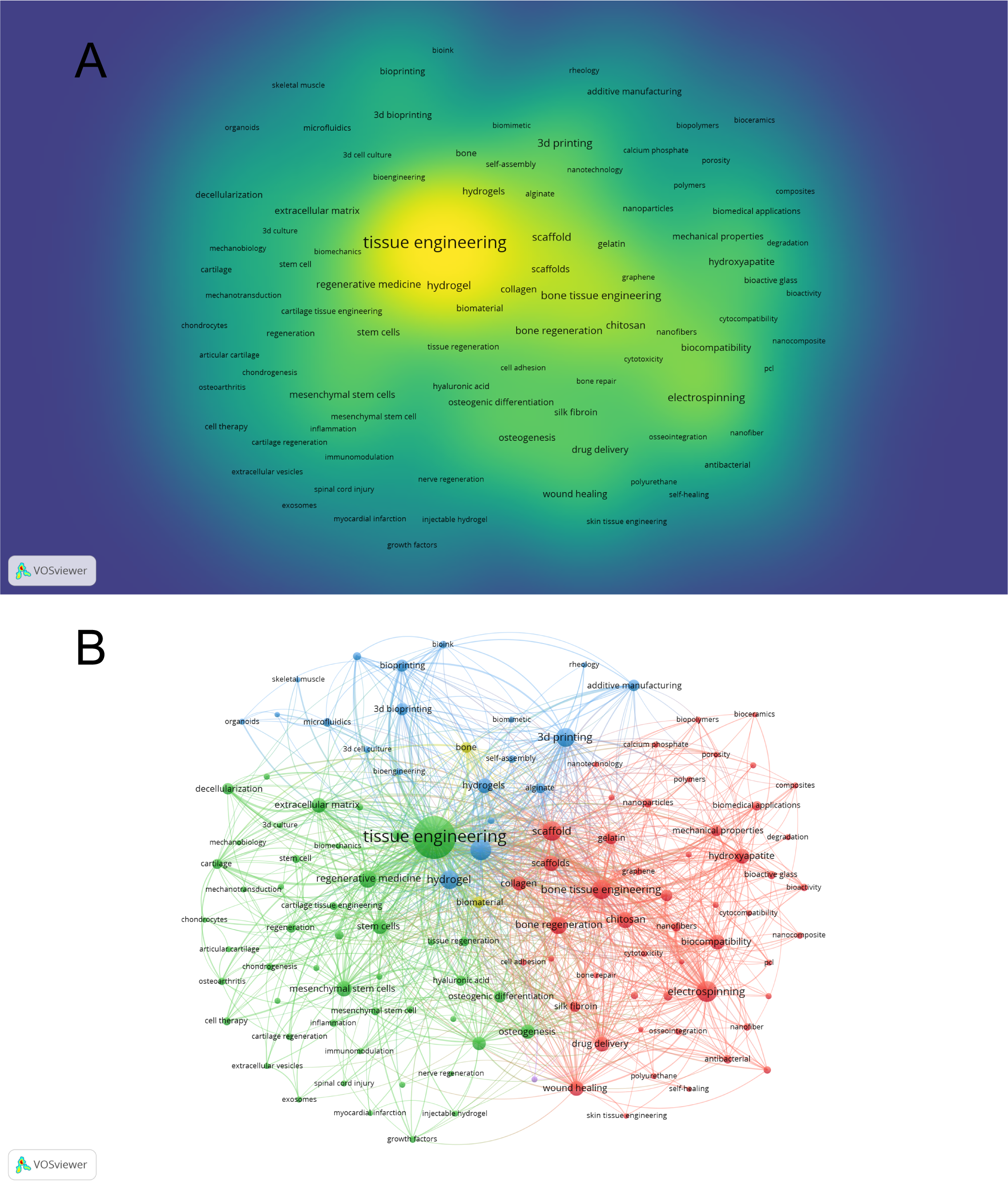

Supplement: Supplementary file 4 [file Image3.PNG]
